# Supplementary material for: Risk of dementia in Nepal: A cross-sectional survey in mountainous, hilly, and lowland regions
Source: PLoS One. 2025 Aug 6;20(8):e0328720. doi: 10.1371/journal.pone.0328720 (PMC12327675; doi:10.1371/journal.pone.0328720)
Supplement: S2 File — (DOCX) [file pone.0328720.s002.docx]

SI Checklist

Inclusivity in global research

**Ethical considerations, permits and authorship**

*This section is applicable to all research types.*

Provide details as to who granted permissions and/or consent for the study to take place in the Methods section of your manuscript. This should include the names of **all** ethics boards, governmental organizations, community leaders or other bodies that provided approval for the study. If individuals provided approval refer to these people by their role or title but do not list their name(s).
If there were any deviations from the study protocol after approval was obtained please provide details of these changes in the Methods section of your manuscript.
Did this study involve local collaborators that are residents of the country where the research was conducted or members of the community studied? If you do not have any authors from said communities, please provide an explanation for this below.

Yes, the study was conducted in collaboration with Ageing Nepal, based in Kathmandu, Nepal. The second author, Sanju Thapa Magar, is a resident of Nepal.

Reported on page number: Reported on page number: Ethical approval from University of Huddersfield (SREIC/2021/090) and Nepal Health Research Council (Ref: 1303) is provided in page 6 under **Ethical consideration**

Everyone listed as an author should meet PLOS’ criteria for authorship and all individuals who meet these criteria should be included in the author byline, rather than the acknowledgements. For further information please see the journal’s Authorship Policy.

**Human subjects research (e.g. health research, medical research, cross-cultural psychology)**

Did you obtain written informed consent from a representative of the local community or region before the research took place? How did you establish who speaks for the community? Details of written informed consent obtained from study participants should be reported separately in the Methods section of your manuscript.

Yes, we obtained ethical clearance from the Nepal Health Research Council prior to data collection. Informed consent was obtained from all research participants, and this process is detailed in the ethical considerations subsection within the Methods section of the manuscript.

How did members of the local community provide input on the aims of the research investigation, its methodology, and its anticipated outcome(s)?

The project was designed in collaboration with the second author, Sanju Thapa Magar, CEO of Ageing Nepal, who has over 10 years of experience working with older people in Nepal. Additionally, during both the project design phase and the training of enumerators, we consulted and engaged local dementia experts with direct experience supporting individuals living with dementia. Their contributions are acknowledged in the manuscript.

When engaging with the local community, how did you ensure that the informed consent documents and other materials could be understood by local stakeholders?

To ensure that informed consent documents, including the participant information sheet, and other materials were easily understood by local stakeholders, we adopted a culturally and linguistically sensitive approach. All materials were translated into the local language (Nepali), with translations reviewed by native speakers familiar with both research terminology and community contexts. Verbal explanations in plain language were provided during the consent process, allowing participants to ask questions and seek clarification. Enumerator training emphasized clear communication to ensure participants fully understood the study’s purpose, their rights, and what participation involved. Understanding was also monitored and confirmed during data collection.

Will the findings of the research be made available in an understandable format to stakeholders in the community where the study was conducted (e.g. via a presentation, summary report, copies of publications, etc.)? Please provide details of how this will be achieved.

Yes, the findings of the research will be made available to local community in an accessible and understandable format. Ageing Nepal will facilitate the disseminate the results in the local language through community presentations, and other appropriate formats to ensure clear communication and engagement with the local population.
