# Supplementary material for: Risk of dementia in Nepal: A cross-sectional survey in mountainous, hilly, and lowland regions
Source: PLoS One. 2025 Aug 6;20(8):e0328720. doi: 10.1371/journal.pone.0328720 (PMC12327675; doi:10.1371/journal.pone.0328720)
Supplement: Attachment — Submitted filename: Manuscript ID -PONE-D-24-59676.docx [file pone.0328720.s003.docx]

| Manuscript ID  Title | PONE-D-24-59676  Risk of Dementia in Nepal: A Cross-Sectional Survey in Mountainous, Hilly, and Lowland Regions |
| --- | --- |
| Comments | Thank you for the opportunity to read your work. Overall, this is an interesting and relevant paper aimed to assess dementia risk in Nepal and compare differences based on age, gender and geographic locations.  However, there are a few methodological and minor concerns regarding the methods and results sections. There is an assumption that readers understand the Nepal’s geography, hence very little rationale was presented to support the study aims. It would be helpful to put the study in context for readers to understand the findings. The analysis method is appropriate, although clarification of a few details will be beneficial. The discussion needs an in-depth analysis of the results which will clarify and facilitate understanding of the findings and their implications on dementia risk assessment. Please see the detailed comments attached for your consideration. |
| Introduction | In line 58, *Various factors have been reported to be associated with dementia in the literature;* Do authors mean the risk of dementia? Please be specific.  In line 76: authors provide evidence throughout the introduction to support risk factors including age and gender for dementia risk assessment. However, it is unclear why geographical location is considered a risk factor to be assessed in this study. Please consider elaborating this a bit more for clarity. For international readers, it would be beneficial to provide a context of the Nepalese regions and a rationale for why the study has to be conducted in the regions with Hilly, mountainous and low lying areas.  **Aim:** There is an assumption that geographic location may be a contributory factor to the risk of dementia. However, this is not clearly stated or supported in the introduction. Thus, comparing the prevalence in three geographic regions is unclear. I would be cautious to use the word prevalence if the diagnosis of dementia or cognitive impairment is not confirmed. Perhaps dementia risk will be most appropriate.  In line 85: consider reframing the objective without the statistical analysis. |
| Design & Methods | There is an assumption that readers know regions in Nepal. How many regions are in Nepal? How many of these regions are in the Hilly, mountainous and low-lying regions? Without the context, it is challenging to comprehend how the stratified sampling was done.  How was the random selection of districts done. How many districts are there in a region? Did authors use a sampling frame for the stratification? If so, how was the frame developed?  My understanding from the methods section is that this was a three-stage stratified sampling process. First, regional sites, district and municipality selections. However, it is not clear how older adults 60+ years were selected. Please make clear how the participants were recruited for the study. What was the inclusion criteria for older adults participating in the study? Did older adults with mild and moderate cognitive impairment participate in the study?  **Survey instrument:** Please elaborate a bit about the Rowland Universal Dementia Assessment Scale (RUDAS) tool. What items are used to measure prevalence of cognitive impairment? Is this a Likert or a dichotomous scale. Is the tool unidimensional or does it have domains? How is the tool rated and summed? How are the scores categorized eg mild, moderate or severe cognitive impairment. What is the reported reliability of the tool? How was the Kobo Toolbox used for data collection. Please clarify to facilitate understanding.  Was the data collected in a different language than English?  What informed the sample size of 933? Or how did authors attain a sample size of 933?  Line 126-130 should be moved to the methods and not statistical analysis sections.  Consider elaborating a bit more about how the statistical analyses were done. Eg How many variables were entered for the logistic analysis? What variable(s) was the outcome variable? |
| Ethical approval | In line 142: Please check spelling |
| Results | While the findings are interesting, the results are a bit challenging to comprehend given the limited description of the survey tools. |
| Discussions | It would be interesting to see the specific variations in geographic location on dementia risk in Nepal.  In line 190: the 75% prevalence of dementia symptoms were with older adult residents in LTC. Your study was conducted with community dwelling older adults. Please elaborate a bit about this study outcome. Consider other factors that could account for the high prevalence of dementia symptoms in residential homes compared to your finding.  In line 193: what was the result of the study and what does the finding mean for your result?  **Strength & limitations:** This is confusing given that item description was inadequately explained in the methods section. If an elaborate description of the scale was presented earlier, it would have made sense why the scale adaptation would be a strength or limitation.  In line 250: I was curious to see what could account for the geographic variations in dementia risk especially for older adults in Nepal. However, supporting your findings with rurality in general, maybe inadequate to highlight the specific dementia risk for older adults in these geographic locations in Nepal. If there are no supporting evidence for dementia risk in specific locations, like hilly, mountainous and low-lying areas, it would be worthwhile to reframe the topic and objectives in line with rurality. |
| Conclusion | While there were significant dementia risk variations in geographic locations, I wondered what specific tailored care could be developed for older adults in mountainous regions compared with those in low lying regions. |
